# Reduction of nitroarenes by magnetically recoverable nitroreductase immobilized on Fe_3_O_4_ nanoparticles

**DOI:** 10.1038/s41598-020-59754-1

**Published:** 2020-02-18

**Authors:** Qikun Zhang, Liping Yu, Baoliang Liu, Fulin Li, Bo Tang

**Affiliations:** 1 College of Chemistry, Chemical Engineering and Materials Science, Key Laboratory of Molecular and Nano Probes, Ministry of Education, Collaborative Innovation Center of Functionalized Probes for Chemical Imaging in Universities of Shandong, Shandong Normal University, Jinan, 250014 P. R. China; 2 Water Resources Research Institute of Shandong Province, Jinan, 250014 P. R. China

**Keywords:** Catalyst synthesis, Synthesis and processing

## Abstract

Enzymes as catalysts have attracted significant attention due to their excellent specificity and incomparable efficiency, but their practical application is limited because these catalysts are difficult to separate and recover. A magnetically recoverable biocatalyst has been effectively prepared through the immobilization of a nitroreductase (oxygen-insensitive, purified from *Enterobacter cloacae*) onto the Fe_3_O_4_ nanoparticles. The magnetic nanoparticles (MNPs) were synthesized by a coprecipitation method in an aqueous system. The surfaces of the MNPs were modified with sodium silicate and chloroacetic acid (CAA). Using 1-ethyl-(3-dimethylaminopropyl) carbodiimide hydrochloride (EDC) through a covalent binding, nitroreductase was loaded onto the modified magnetic carriers through covalent coupling, and thus, a magnetically recoverable biocatalyst was prepared. The free and immobilized nitroreductase activity was also investigated by the reduction of p-nitrobenzonitrile using nicotinamide adenine dinucleotide phosphate (NAPDH) as a cofactor. The activity of the immobilized enzyme was able to maintain 83.23% of that of the free enzyme. The prepared enzyme can easily reduce substituted nitrobenzene to substituted aniline at room temperature and atmospheric pressure, and the yield is up to 60.9%. Most importantly, the loaded nitroreductase carriers can be easily separated and recycled from the reaction system using an externally applied magnetic field. The magnetically recoverable biocatalyst can be recycled and reused 7 times while maintaining high activities and the activity of the magnetic catalyst can be maintained at more than 85.0% of that of the previous cycle. This research solves the recovery problem encountered in industrial applications of biocatalysts and presents a clean and green method of preparing substituted aniline.

## Introduction

Biocatalysis is an important green and sustainable technology, because it is an ideal approach that can be used to meet the challenges of energy conservation and environmental sustainability^1,2^. Enzymes, which are natural biocatalysts, are becoming more and more important in industrial applications, because enzymes can be used at pH values, temperatures and pressures that are moderate, and enzymes form few byproducts and have high activities and an unparalleled selectivity^3–5^. However, there are two challenges that need to be addressed in practical applications. One of the challenges is that enzymes can be denatured, i.e., the native three-dimensional structure unfolds, and this occurs as a result of exposure of the enzyme to solvents or changes in the temperature and pH. Another challenge is that enzyme reuse is often hardly feasible. The recovery and reuse of enzymes is also a decisive factor in the production cost^6^.

Specifically, enzymes lack long term stability under processing conditions, and it is very difficult to recover the enzyme from the reaction system. Most heterogeneous systems require either a filtration or centrifugation step or tedious processing of the final reaction mixture to recover the enzyme^7,8^. In order to solve the problem of recovery and application of biological enzyme catalyst, it is a feasible and popular strategy to immobilize it on an ideal support. These supports include porous or nonporous materials^9–13^. They can be inorganic materials^14–21^, organic materials or polymer functional materials^22–24^. Moreover, the size of the support significantly affects the catalytic mechanism and efficiency of the final catalyst. For example, the use of nanoparticle supports is of particular interest, nanoscale support may avoid the entrapment of active sites caused by porous supports and offer a better solution for diffusion problems that are often encountered in conventional reaction systems^5,6^. However, it is difficult to separate and recover from the reaction system conveniently using nanoparticles as the carrier of enzyme catalyst.

Magnetic nanoparticles (MNPs) can be used for numerous applications because of their interesting properties^25–35^. One of the most attractive features of MNPs is their separation capabilities. Thus, we reasoned that separation of nitroreductase from the reaction system and its re-use could be easily achieved by using magnetically separable nanoparticles. Magnetically recoverable catalysts can be utilized in a wide range of reactions. Magnetic nanoparticle surfaces can be tailored for a specific application by designing a specific surface activation, thus resulting in versatile carriers^36–41^. Developments in the immobilization of enzymes can improve the stability and activity of enzymes and expand the range of conditions in which the enzymes can be used by significantly increasing the total turnover number and significantly improving the overall process efficiency^42^.

Chloroanilines (CAs) are among the most important classes of compounds used in organic synthesis^43–45^. The hydrogenation of aromatic nitro compounds to aromatic amines is the most common method used to prepare CAs. However, currently used industrial hydrogenation processes still have drawbacks: high temperatures are required, and organic solvents that are toxic, flammable, and environmentally hazardous are used. Hydrogenation catalyzed by nitroreductase is now considered to be the most promising method for the preparation of chloroanilines^46^.

Herein, MNPs were prepared effectively by a coprecipitation method in a water system. The MNPs were modified with sodium silicate and CAA. Then, 1-ethyl-(3-dimethylaminopropyl) carbodiimide hydrochloride (EDC) was used as a covalent coupling agent, and nitroreductase was loaded onto the modified magnetic carriers. Therefore, a magnetically recoverable biocatalyst was prepared. The magnetic properties of the magnetically recoverable enzyme were characterized by Vibrating Sample Magnetometry (VSM). The magnetically recoverable enzyme was used to reduce o-chloronitrobenzene.

## Experimental

All of the chemicals used in this work were of pure analytical grade, and the solutions were prepared with distilled water. Sodium silicate (Na_2_SiO_3_), chloroacetic acid (CAA), sodium nitrite (NaNO_2_), ammonium sulfamate (NH_4_SO_3_NH_2_) and sulfuric acid (H_2_SO_4_) were purchased from Shanghai Sinopharm Chemical Co., Ltd. EDCand N-hydroxysuccinimide (NHS) were purchased from Shanghai Civic Chemical Technology Co., Ltd. N-(1-naphthalene) ethylenediamine hydrochloride (C_12_H_14_N_2_∙2HCl) was purchased from Tianjin No.3 Chemical Plant. o-Chloroaniline (C6H6ClN) was purchased from Beijing Aoboxing Bio-Tech Co., Ltd. Nitroreductase was obtained from Shandong Dongdu Food Co., Ltd. The temperature and pH for the optimal activity of the enzyme are 80–90 °C and 5.5–7.0.

### Preparation of the Fe_3_O_4_ magnetic nanoparticles

The Fe_3_O_4_ nanoparticles were prepared by a coprecipitation method^47,48^. In a typical process, 1.7 g FeCl_3_∙6H_2_O and 0.66 g FeCl_2_∙4H_2_O (the molar ratio of the two materials was 2:1) were placed in a 250 ml 3-neck flask, and 20 ml deionized water was added to the flask. The solution was stirred, and the flask was purged with N_2_. The flask was then placed in a water bath at 90 °C, and ammonium hydroxide (1.5 M) was added dropwise. The color of the mixture gradually changed from orange-yellow to orange-red gradually and eventually became black, and a black precipitate was simultaneously obtained. When the pH increased to 8.0–9.0, an additional 10 ml ammonium hydroxide (1.5 M) was added to ensure complete hydrolysis. The stirring was stopped, and magnets were placed under the 3-neck flask. After the material was separated by the magnets, the black precipitate was redispersed in distilled water, and the top layer of the suspension was removed using a pipette. The resulting black particles were washed 3 times with deionized water and were then dispersed and stored in deionized water (0.1 g·ml^−1^) for later use.

### Preparation of the silica-coated magnetic nanoparticles

The Fe_3_O_4_ nanoparticles (0.5 g) were placed in a 250 ml 3-neck flask, and 0.6 g Na_2_SiO_3_∙9H_2_O and 100 ml deionized water was added to the flask. The solution was stirred, and the flask was purged with N_2_. The mixture was heated to 80–90 °C. Hydrochloric acid was added dropwise to adjust the pH to 6.0–7.0. The flask was then placed in a water bath at 90 °C for 60 min under N_2_. The stirring was stopped, and magnets were placed under the 3-neck flask. After the material was separated by the magnets, the black precipitate was redispersed in distilled water, and the top layer of the suspension was removed with a pipette. The resulting black particles were washed with 100 ml deionized water and separated by magnetic decantation, and this process was repeated 3 times. The silica-coated Fe_3_O_4_ nanoparticles were dispersed in deionized water (0.1 g · ml^−1^) for later use.

### Preparation of the ClCH_2_COOH-coated magnetic nanoparticles

The silica-coated Fe_3_O_4_ magnetic nanoparticles (0.3 g) and 100 ml deionized water were added to a 250 ml 3-neck flask. The solution was stirred, and the flask was purged with N_2_. The flask was then placed in a water bath at 50–60 °C. Then, 0.75 g Na_2_CO_3_ and 0.4 g ClCH_2_COOH were added to the mixture. The reaction was allowedto proceed for 1.0–1.5 h. The stirring was stopped, and a magnet was placed under the 3-neck flask. After the material was separated by a magnet, the black precipitate was redispersed in distilled water, and the top layer of the suspension was removed with a pipette. The resulting black particles were washed 3 times with 100 ml deionized water. The modified Fe_3_O_4_ nanoparticles (ClCH_2_COOH@SiO_2_@Fe_3_O_4_) were dispersed and stored in deionized water (0.1 g·ml^−1^) for later use.

### Immobilization of nitroreductase on the modified magnetic nanoparticles

**Figure 1:**
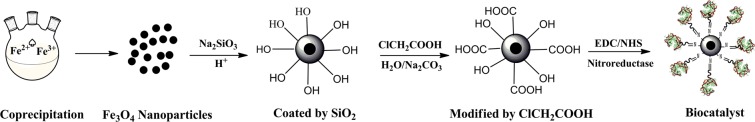
Scheme of the preparation of the magnetic biocatalyst.

A dispersion was prepared by adding 0.1 g modified magnetic nanoparticles to 100 ml phosphate-buffered saline (PBS, pH = 7.0). 100 μl of EDC were added to 1 ml of the solution. The mixtures were dispersed for approximately 5 min using an ultrasonic oscillator. Then, 100 μl NHS was added to the abovementioned mixture. 50 μl of nitroreductase was also added to the abovementioned solution. The solution was placed in a constant temperature incubator and shaken for 2.0 h. The mixture was removed from the incubator and diluted to 1.0 mg·ml^−1^ by magnetic decantation. The resulting dispersion was stored at 4 °C in a refrigerator. Figure 1 illustrates the typical procedure for the preparation of the magnetic biocatalysts.

### Physical and chemical characterization

#### Transmission electron microscopy

Transmission electron microscopy (TEM) was used to characterize the appearance and size of the bare Fe_3_O_4_ nanoparticles, the surface-modified nanoparticles and the nanoparticles loaded with nitroreductase. A typical characterization process is as follows. An appropriate amount of the sample was placed into a PE tube containing anhydrous ethanol. The sample was dispersed by an ultrasonic oscillator for 5 min. The dispersion was added to a copper grid and placed under an infrared lamp to volatilize the excess ethanol. The copper grid was inserted into a transmission electron microscope, and the sample was observed and imaged using a voltage of 200 kV.

#### Infrared spectroscopy analysis

Infrared spectroscopy is a powerful tool for the identification of functional groups present in organic compounds. In this technique, an absorption spectrum is produced and shows the transition of vibrational and rotational energy levels of atoms in molecules when infrared light is absorbed by the substances. In the experiment, 1.0–2.0 mg dry samples and 50–100 mg dry potassium bromide crystals (powder) were ground in an agate mortar to form a fine powder with particle sizes less than 2.0 µm. This powder was then mixed evenly. The resulting powder was loaded into a mold and pressed into sheets on an oil press, and the sheets were then tested by an infrared spectrometer. The resolution and the detection range of the infrared spectrometer were 4.0 cm^−1^ and 500–4000 cm^−1^, respectively. Air was used as the blank, and the scanning times were set to 32.

#### X-ray diffraction analysis

The XRD spectra were obtained using a D8 powder diffractometer, which was used to characterize the change in the crystallinity. The diffractometer used Cu Kα radiation, a 30 kV tube voltage, a tubecurrent of 20 mA, a scanning speed of 5°/min, and a scanning range of 2*θ* = 20° ~ 90°.

#### Magnetic characterization of the nanoparticles

The magnetic properties of the bare and modified Fe_3_O_4_ nanoparticles and the Fe_3_O_4_ nanoparticles loaded with nitroreductase were measured by a vibrating sample magnetometer (VSM).

### Determination of the enzymatic activity

**Figure 2:**
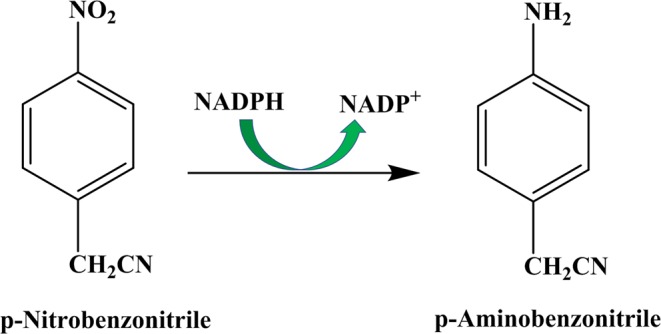
Chemical scheme of the enzymatic assay.

The nitroreductase activity was determined based on the change in the absorbance of NAPDH at 340 nm. A typical process for the determination of the enzyme activity (for a 1.0 ml total volume) was as follows: 970 µl PBS (pH 7.0, 0.1 M) and 10 µl p-nitrobenzonitrile (100 mM) were added to a 1 mL quartz cuvette. After the contents of the cuvette were mixed, the absorbance at 340 nm was adjusted to zero. Then, 10 µl NAPDH (10 mM) and 10 µl of a dilute nitroreductase enzyme solution (1.0 mg·ml^−1^) were added to the solution. The changes in the absorbance at 340 nm were measured at one-minute intervals. Figure 2 illustrates the chemical scheme of the enzymatic assay.

The enzyme activity is defined, using the abovementioned conditions, as the amount of enzyme required to catalyze the oxidation of 1.0 µmol NADPH per minute or the reduction of 1.0 µmol NADP^+^ per minute.

The enzyme activity can be calculated as follows:

1 $$({\rm{U}})=\frac{CAB\times V\times {10}^{3}}{6220\times {10}^{3}}$$

where *CAB* is the change in the absorbance at 340 nm after 1 min, *V* is the volume of the reaction solution, 6220 is the molar extinction coefficient (l·mol^−1^cm^−1^), and *l* is the optical distance (cm).

The specific enzyme activity can be calculated as follows: Specific activity (U/g) = enzyme activity U/weight of nitroreductase (g).

### Reduction of o-chloronitrobenzene to o-chloroaniline and product isolation

**Figure 3:**
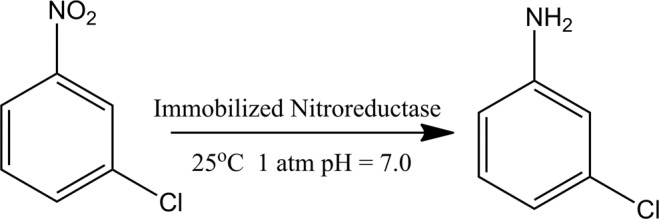
Scheme the production of o-chloroaniline by magnetic biocatalyst.

The magnetic catalyst was used to produce o-chloroaniline from o-chloronitrobenzene (Fig. 3). A typical reaction process is as follows (where the total volume is 5.0 ml, and o-chloronitrobenzene is used as an example): 400 μl PBS (0.10 M, pH 7.0), 50 μl (1.0 mg·ml^−1^) of the loaded nitroreductase dispersion solution, and 50 μl NAPDH (10 mM) were added to a 5.0 ml EP tube. Then, 1.0 ml (0.5 wt%) of an o-chloronitrobenzene solution was added. Finally, the whole system was supplemented to a total volume of 5.0 ml with deionized water. The EP tube was placed in a constant temperature incubator. After the reaction proceeded at room temperature and atmospheric pressure for 0.5–2.0 h, the reaction was stopped by heating. The magnetic catalyst was isolated using an external magnet. The solvent containing reaction product was removed in vacuo. The corresponding aniline was purified by column chromatography (silica; n-hexane-ethyl acetate mixture). Then, dried over anhydrous Na_2_SO_4_ and the solvent was removed in vacuo. Herein, we used colorimetry to determine the total aromatic amino content in the products to calculate the actual output of chloroaniline. These results were compared with the theoretical yield to obtain the catalytic reaction rate for the production of the chloroaniline.

### Determination of chloroaniline

**Figure 4:**
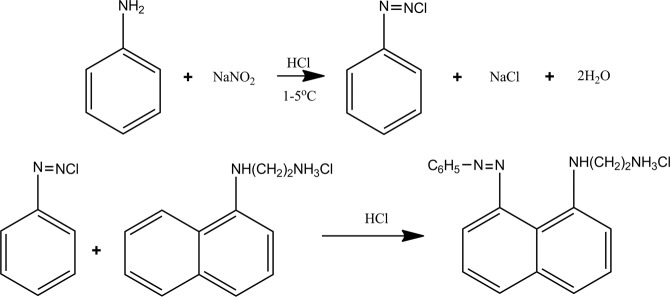
Scheme the determination of aniline content in reaction with a colorimetric method.

The concentration of aniline in the reaction system was determined by a colorimetric method. The aniline concentration can be determined because aniline in the solution reacts with sodium nitrite to generate a diazo compound, which couples with N-(1-naphthalene) ethylenediamine diazo hydrochloride to produce a magenta color (Fig. 4). The absorbance of the solution at 545 nm is measured by a spectrophotometer to determine the concentration of aniline.

To obtain a standard curve, seven solutions with different concentrations of o-chloroaniline were prepared precisely. Thevolumes of the o-chloroaniline standard solutions (0.5 wt%) were 0.00, 0.25, 0.50, 1.00, 2.00, 3.00, and 4.00 ml. The solutions were supplemented with deionized water to achieve a final volume of 10 ml. The determinations were performed according to the protocol described in 2.6. A standard curve was plotted with the o-chloroaniline concentration (mmol·l^−1^) on the horizontal axis (X) and the absorbance on the vertical axis (Y). The standard curve can be fit with Y = 0.012665 + 0.337X, and the linear correlation coefficient of the fitted standard curve is 0.99999. The detection limit of this method is 0.03 mmol·l^−1^.

To calculate the of product yield (Y_R_, %), the following formula was used:

2 $${Y}_{R}=\frac{X\times {M}_{P}157.56}{Mr\times 127.57}\times 100 \% $$

where X is the content of pure chloroaniline reaction product (%), Mp is the quantity of reaction products (g), and Mr is the quantity of the reactant (g).

## Results and Discussion

### TEM characterization of the magnetic nanoparticles

**Figure 5:**
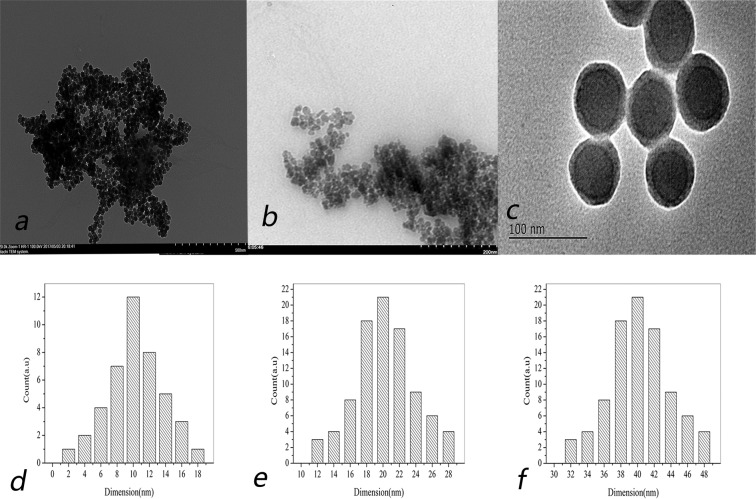
TEM images and diameter of the bare (**a,d**), silica- and CA-coated Fe_3_O_4_ nanoparticles (**b,e**) and the final biocatalysts (**c,f**).

The scanning electron microscope images and particle size distributions of the bare magnetic nanoparticles, coated magnetic nanoparticles and final magnetic catalyst are shown in Fig. 5. As shown by the figure, the bare magnetic nanoparticles were evenly distributed, and the distribution indicates that the nanoparticles are monodisperse. The average diameter of the bare particles is approximately 10 nm. The surface of the loaded nanoparticles is much rougher than that of the bare nanoparticles. The average diameter of the coated nanoparticles was larger than that of the bare nanoparticles and was determined to be approximately 20 nm. The final magnetic catalyst has a core-shell structure, and its average diameter is approximately 40 nm.

### Infrared spectroscopy of the magnetic nanoparticles

**Figure 6:**
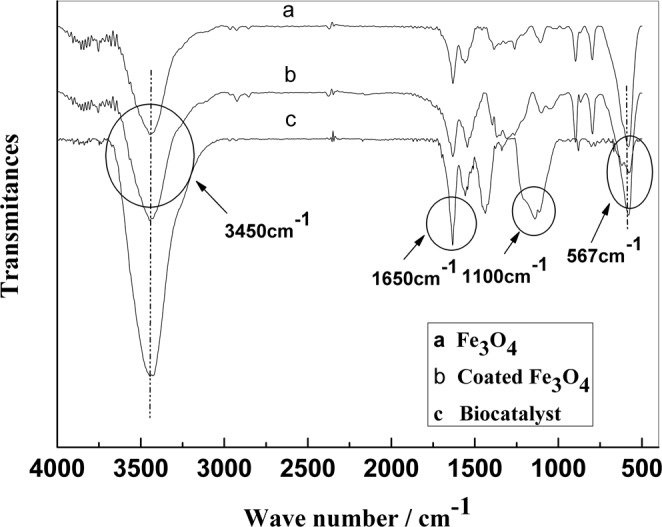
FT-IR spectra of the bare and coated Fe_3_O_4_ nanoparticles and the biocatalyst.

Figure 6 shows the infrared spectrum obtained for the bare Fe_3_O_4_ nanoparticles. In the figure, the absorption peak at 567 cm^−1^ corresponds to the vibration of the Fe–O bonds in the crystalline lattice of Fe_3_O_4_. Compared with the bare magnetic nanoparticles, new bands were present when the magnetic Fe_3_O_4_ nanoparticles were coated with silica and chloroacetic acid. The absorption peak at 3450 cm^−1^ corresponds to the stretching vibration of the O–H bonds in the ClCH_2_COOH structure. The absorption peak at 1650 cm^−1^ corresponds to the stretching vibration of C–O, while the absorption peak at 1100 cm^−1^ corresponds to the asymmetric stretching vibration absorption peak of Si–O–Si (Fig. 2b). Figure 2c shows the infrared spectrum obtained for the magnetic catalyst. The figure shows that the intensity of the absorption peak at 3450 cm^−1^ increases significantly, as a result of the stretching vibration of -NH_2_ and indicates that nitroreductase was immobilized successfully on the magnetic nanoparticles.

### X-ray diffraction analysis of the magnetic nanoparticles

**Figure 7:**
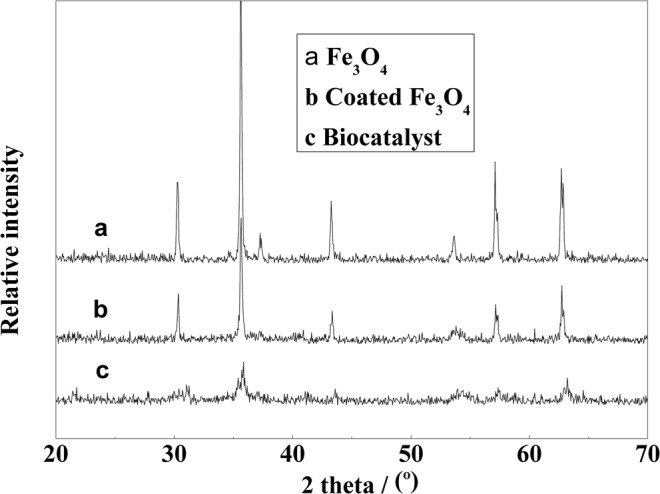
The X-ray diffraction patterns obtained for the bare and coated Fe_3_O_4_ nanoparticles.

The X-ray diffraction (XRD) patterns obtained for the bare and coated magnetic nanoparticles and the final magnetic catalyst are shown in Fig. 7. According to the Joint Committee on Powder Diffraction Standards (JCPDS) database, the XRD pattern of a typical Fe_3_O_4_ crystal with a spinel structure has six characteristic peaks at 2θ = 30.1°, 35.5°, 43.1°, 53.4°, 57.0°, and 62.6°, which are attributed tothe (2 2 0), (3 1 1), (4 0 0), (4 2 2), (5 1 1), and (4 4 0) crystallographic planes of the Fe_3_O_4_ structure, respectively. As shown in Fig. 3, the XRD pattern obtained for the bare Fe_3_O_4_ nanoparticles has distinct crystalline peaks at 30.35°, 35.69°, 43.25°, 53.70°, 57.04° and 62.81°. The XRD patterns obtained for the bare and coated magnetic nanoparticles and the final magnetic catalyst were consistent with the pattern expected for magnetite. Therefore, the modification treatment did not cause the crystalline structure of Fe_3_O_4_ to change. The magnetite nanoparticles modified with SiO_2_ and chloroacetic acid were also found to have a spinel structure. New peaks corresponding to SiO_2_ and chloroacetic acid, which are both amorphous,were not detected in the XRD patterns obtained for the coated magnetic nanoparticles. However, the crystallinity of the samples decreased. This likely occurred because the SiO_2_ and chloroacetic acid coatings partial disrupted the regularity of the Fe_3_O_4_ crystal structure. The results also indicated that some chloroacetic acid molecules were introduced into the Fe_3_O_4_ crystal structure during the coating process.

### Magnetic characterization of the magnetic nanoparticles

**Figure 8:**
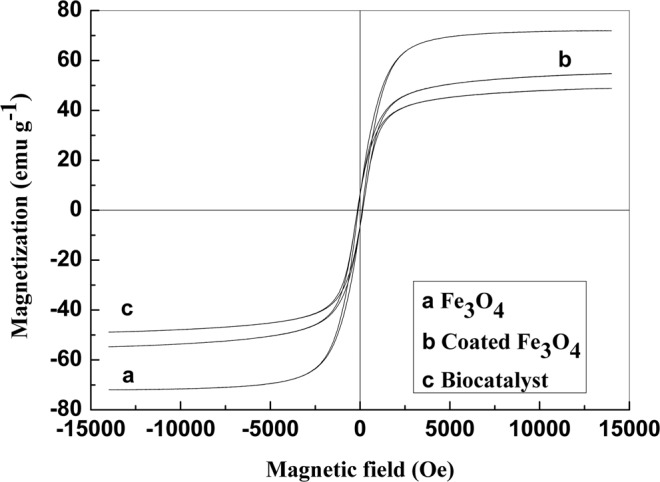
Magnetization curves obtained for the magnetic particles.

The magnetic properties of the bare and coated Fe_3_O_4_ MNPs and the biocatalyst nanoparticles were determined by VSM. Figure 8 shows the corresponding magnetization curves. No coercivity was observed in the magnetization curve obtained for the bare Fe_3_O_4_ MNPs. The saturation magnetization (M_s_) of the bare Fe_3_O_4_ nanoparticles was 71.9 emu·g^−1^, which indicates that the prepared particles exhibited super paramagnetic behavior. Furthermore, changes in the M_s_ of the particles after coating with SiO_2_ and chloroacetic acid were observed. The M_s_ of the bare MNPs decreased from 71.9 emu·g^−1^ to 54.7 emu·g^−1^ after coating with SiO_2_ and chloroacetic acid, and the M_s_ of the magnetic biocatalyst decreased to 48.8 emu·g^−1^. This decrease was attributed to the contribution of the SiO_2_, chloroacetic acid and nitroreductase shells, which are nonmagnetic, to the total mass of the particles. The relative amount of Fe_3_O_4_ present in the composite particles decreases after coating and the immobilization process, which causes the specific saturation magnetization to decrease and the magnetic properties to significantly weaken.

### Activity of nitroreductase

Determination of the enzymatic activity was carried out according to 3.6. All tests were repeated three times. The result of activity of free and immobilized nitroreductase was shown in Table 1. From Table 1, we can see that the activity of nitroreductase decreases after it was loaded onto the Fe_3_O_4_ magnetic nanoparticles. However, the activity of the magnetic catalyst remained at 83.23% of that of the free enzyme. The loss of the enzyme activity of the immobilized nitroreductase enzyme was acceptable, which proves that the loading strategy investigated in this work is feasible.

**Table 1:** Activity of free and immobilized nitroreductase^a^.

items	NR free	NR imm	AR
CAB	0.0030	0.0025	—
V	1	1	—
EC	6220	6220	—
L	1.5	1.5	—
EA	3.22 × 10^−4^	2.68 × 10^−4^	83.23%
SEA	32.2 ± 3	26.8 ± 2	83.23%

^a^Where *NR free* is the Free nitroreductase, *NR imm* is the Immobilized nitroreductase, *AR* is the Activity Recovery (%), *CAB* is the Change of absorbance, *V* is the Volume of reaction system (ml), *EC* is the Extinction coefficient (l·mol^−1^cm^−1^), *L* is the Optical path distance (cm), *EA* is the Enzyme activity (U) and *SEA* is the Specific enzyme activity (U·g^−1^).

### Enzymatic synthesis of aniline

The aromatic amine concentration was determined using a spectrophotometric method with modifications^49^. In a typical process, 1 ml of the supernatant obtained from the aromatic amine reaction solution, which was detailed in 3.8, was added to a 25 ml colorimetric tube. A total volume of 10 ml was achieved by supplementing with deionized water. Hydrochloric acid (1.0 M) was added dropwise to adjust the pH to 1.5–2.0, and a drop of a sodium nitrite solution (5 wt%) was added. The solution was shaken well and allowed to stand for 3 min. Then, 0.5 ml of an ammonium sulfamate solution was added to the abovementioned solutions, and the resulting solutions were sonicated for 3 min. After this, 1.0 ml of a N-(1-naphthalene) ethylenediamine hydrochloride solution was added to the abovementioned mixtures, and 25 ml deionized water was added to the resulting solutions. The solution was shaken well and allowed to stand for another 3 min. Water was used as a blank, and the absorbance of the solution was measured at 545 nm. The concentration of m-chloroaniline was calculated using the standard curve. The test results are shown in Table 2.

**Table 2:** Overview of the yield of o-chloroaniline obtained by the biocatalysts^a^.

Entry	Catalyst	CNcon	CAabs	CAcon	Y_CA_
1	NRfree	4.976 × 10^−2^	0.020	2.81 ± 0.3 × 10^−2^	56.5
2	NRfree	4.976 × 10^−2^	0.022	3.57 ± 0.4 × 10^−2^	71.7
3	NRfree	4.976 × 10^−2^	0.021	3.19 ± 0.3 × 10^−2^	64.1
4	NRimm	4.976 × 10^−2^	0.028	3.03 ± 0.3 × 10^−2^	60.9
5	NRimm	4.976 × 10^−2^	0.078	2.49 ± 0.2 × 10^−2^	50.0
6	NRimm	4.976 × 10^−2^	0.022	2.23 ± 0.2 × 10^−2^	44.8

^a^Where *NRfree* is the Free nitroreductase, *NRimm* is the Immobilized nitroreductase, *CNcon* is the Chloronitrobenzene concentration (μg·ml^−1^), *CAabs* is the Chloroaniline absorbance, *CAcon* is the Chloroaniline concentration (μg·ml^−1^), *Y*_*CA*_ is the Chloroaniline yield (%). The reaction conditions were as follows: the reaction volume was 5.0 ml, the pH was 7.0, the reaction time was 2.0 h, and the reaction proceeded at room temperature and atmospheric pressure.

The results showed that both free and immobilized nitroreductase could effectively catalyze the reduction of o-chloronitrobenzene to o-chloroaniline. The best catalytic yield of free and immobilized nitroreductase was up to 71.7% and 60.9%, respectively.

### Recovery and reuse of immobilized nitroreductase

The recoverability and reusability of immobilized nitroreductase after repeated magnetic separation and reuse was studied under the same conditions described in the activity assay section. After each enzymatic reaction, the magnetic catalysts were magnetically separated and washed twice with deionized water to remove any remaining substrate and product species before the next experiment.

**Figure 9:**
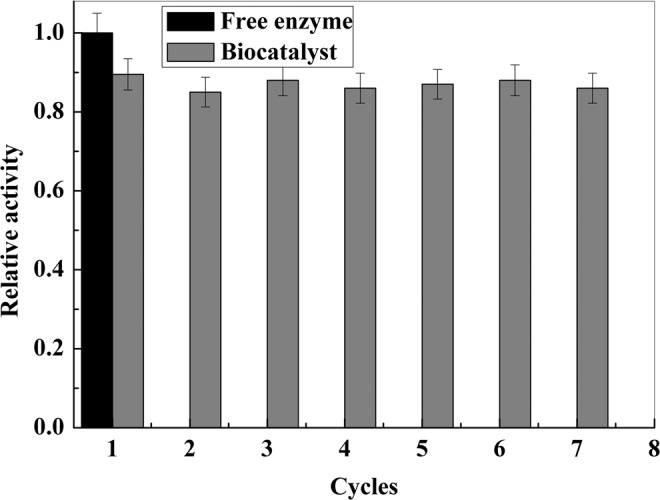
Comparison of free (black) and immobilized nitroreductase (gray). The reaction condition: the temperature and pH are 25 °C and 7.0, and the initial chloronitrobenzene concentration is 49.76 mg·L^−1^.

Figure 9 shows the residual activity of the immobilized nitroreductase enzyme after each cycle. The results indicate that the activities of this enzyme immobilized on the MNPs were excellent, even after several reuses. In this work, the enzyme immobilized on the MNPs can be recycled and reused 7 times while maintaining high activities, and the activity of the magnetic catalyst can be maintained at more than 85.0% of that of the free nitroreductase enzyme.

**Figure 10:**
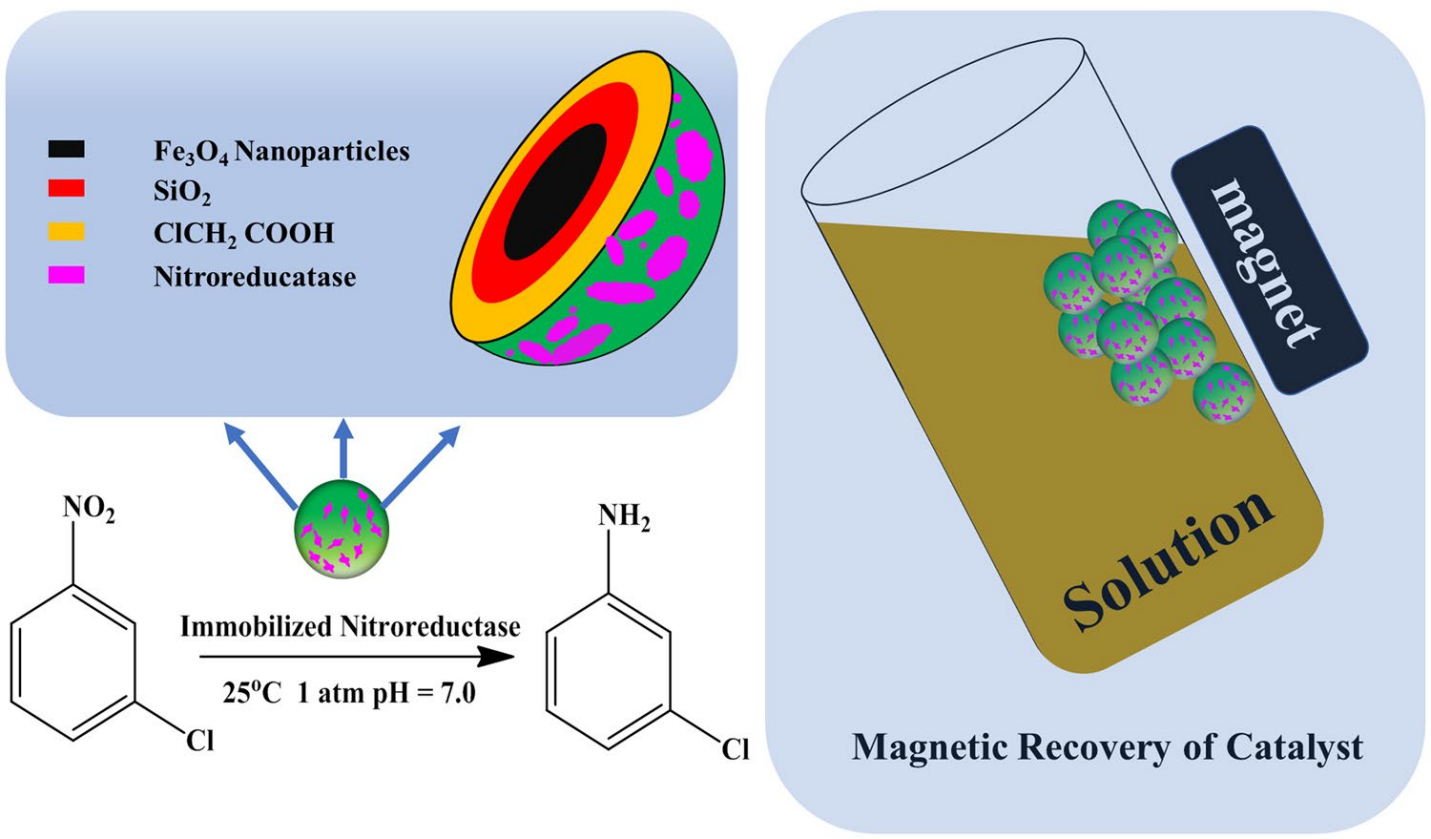
Scheme of reduction nitroarenes by magnetically recoverable nitroreductase immobilized on Fe_3_O_4_ nanoparticles.

Figure 10 shows the scheme of reduction nitroarenes by magnetically recoverable nitroreductase immobilized on Fe_3_O_4_ nanoparticles. Using the prepared magnetic catalyst, the isolation and separation of the enzyme can be easily achieved.

## Conclusions

The Fe_3_O_4_ magnetic nanoparticles were prepared by a coprecipitation method. After chemical modification with sodium silicate and chloroacetic acid, nitroreductase was loaded onto the magnetic carriers by EDC, and thus, a magnetically recoverable biocatalyst was prepared. The activity of immobilized nitroreductase can maintain 83.23% of that of the free enzyme. The prepared biocatalyst can easily reduce o-chloronitrobenzene to o-chloroaniline at room temperature and atmospheric pressure, and the yield is up to 60.9%. The loaded nitroreductase can be easily separated from the reaction system using an applied external magnetic field. After 7 cycles, the activity of the magnetic catalyst can be maintained at more than 85.0% of that of the free nitroreductase enzyme. This work provides a clean and green method to prepare o-chloroaniline.
